# Sexuality, a Fundamental Care Need—Theoretical and Clinical Considerations, a Discursive Paper

**DOI:** 10.1111/scs.70207

**Published:** 2026-02-19

**Authors:** Birgitte Schantz Laursen, Philippa Rasmussen, Siri Lygum Voldbjerg, Mette Grønkjær, Britt Laugesen

**Affiliations:** ^1^ Clinical Nursing Research Unit, Aalborg University Hospital Aalborg Denmark; ^2^ Sexology Centre, Aalborg University Hospital Aalborg Denmark; ^3^ Center for Sexology Research, Department of Clinical Medicine Aalborg University Aalborg Denmark; ^4^ Faculty of Health and Medical Sciences, Adelaide Nursing School Adelaide Australia; ^5^ Department of Clinical Medicine Aalborg University Aalborg Denmark; ^6^ University College North Jutland Aalborg Denmark

**Keywords:** fundamentals of care, nursing, patient‐centred care, sexual dysfunction, sexuality

## Abstract

**Background:**

Sexuality is a crucial component of quality of life yet often remains an under‐recognised and overlooked patient need within nursing care. Despite patients expressing a desire to discuss their sexuality with nurses, significant barriers hinder these conversations, leading to unmet needs and potential negative impacts on well‐being.

**Aim:**

This discursive paper aims to discuss how the Fundamentals of Care (FoC) framework can support nurses in acknowledging and addressing sexuality as a fundamental aspect of patient care.

**Method:**

A broad, structured literature search was conducted in PubMed, CINAHL and Embase (2010–2026) focusing on sexuality, fundamental care needs, nursing theories and the nurse's role in addressing sexual dysfunction. The findings were critically reflected upon using the FoC framework as a lens for understanding and improving practice.

**Findings:**

The analysis reveals that the FoC framework's emphasis on relationship, integration of care and context can facilitate a more holistic and patient‐centred approach to addressing sexuality. Integrating the PLISSIT model (Permission, Limited Information, Specific Suggestions, Intensive Therapy) alongside the FoC framework provides a structured approach to initiating conversations and offering support. Organisational support, clear guidelines and adequate nurse education are also critical for overcoming barriers and fostering a culture where sexuality is openly addressed.

**Conclusion:**

Utilising the FoC framework, supplemented by the PLISSIT model and a supportive organisational context, can empower nurses to acknowledge and address patients' sexual needs, ultimately improving their overall well‐being and breaking down the ‘two‐way taboo’ surrounding sexuality in healthcare.

## 1 Introduction

Cancer and chronic diseases often impact various aspects of a patient's sexuality [1, 2, 3, 4, 5] including sexual dysfunctions, a decline in intimate relationships and overall quality of life [1, 6, 7, 8]. Patients with sexual dysfunctions express the need to discuss their sexuality with nurses and other healthcare professionals and expect nurses to initiate these conversations [2, 5, 9]. Studies underline that effective communication from nurses regarding sexuality can enhance and improve patients' quality of life, highlighting the significance of addressing this issue [7]. Conversely, no or inadequate communication from nurses about sexuality appears to leave patients with unidentified and untreated sexual problems [7, 10, 11].

According to the World Health Organization (WHO), sexuality is an essential and inseparable component of human life that should be addressed in patient care [12]. Sexuality extends beyond mere physiological processes such as erection, lubrication, orgasm and intercourse, encompassing bodily, social and psychological aspects like intimacy, identity, body image, sensuality and affection (ibid). Although sexuality is recognised as a comprehensive biopsychosocial phenomenon and the Danish National Board of Health requires its inclusion in patient documentation, the patient's sexuality remains an under‐recognised and often overlooked patient need in nursing [13]. Only a few nursing theories address patient's sexuality and none explicitly define it as a patient need.

Abraham Maslow's theory of needs, upon which Virginia Henderson—among others—bases her own, posits that sexual needs can arise at various levels in human existence. Sexuality can be a basic physiological need, a social need and part of the need for love, recognition, community and self‐realisation [14]. For Virgina Henderson, sexuality is indirectly addressed within her 14 areas of needs, e.g., within the needs to maintain normal body functions and enter relationships. Joyce Travelbee's theory focuses on relationship and meaning, including intimacy as integral to human well‐being. Finally, Kari Martinsen's theory focuses on ethics and care, wherein respect for the whole person encompasses sexuality [15].

Common factors that impede conversations about sexual dysfunctions with patients include sociocultural norms, a lack of routine inquiry, insufficient priority, time constraints and inadequate organisational support [16, 17, 18]. Furthermore, sexuality is a sensitive topic often characterised by a two‐way taboo where negative experiences of professional capacity and absence of institutional policies and guidelines hinder nurses from addressing patients' sexual health issues in their professional practice [17]. To bridge the gap between patients' needs for conversations about sexuality and to increase nurses' awareness of the responsibility in addressing these needs, the aim of this discursive paper is to discuss how to assist nurses in acknowledging and addressing sexuality as a fundamental aspect of patient care.

## 2 Background

**FIGURE 1:**
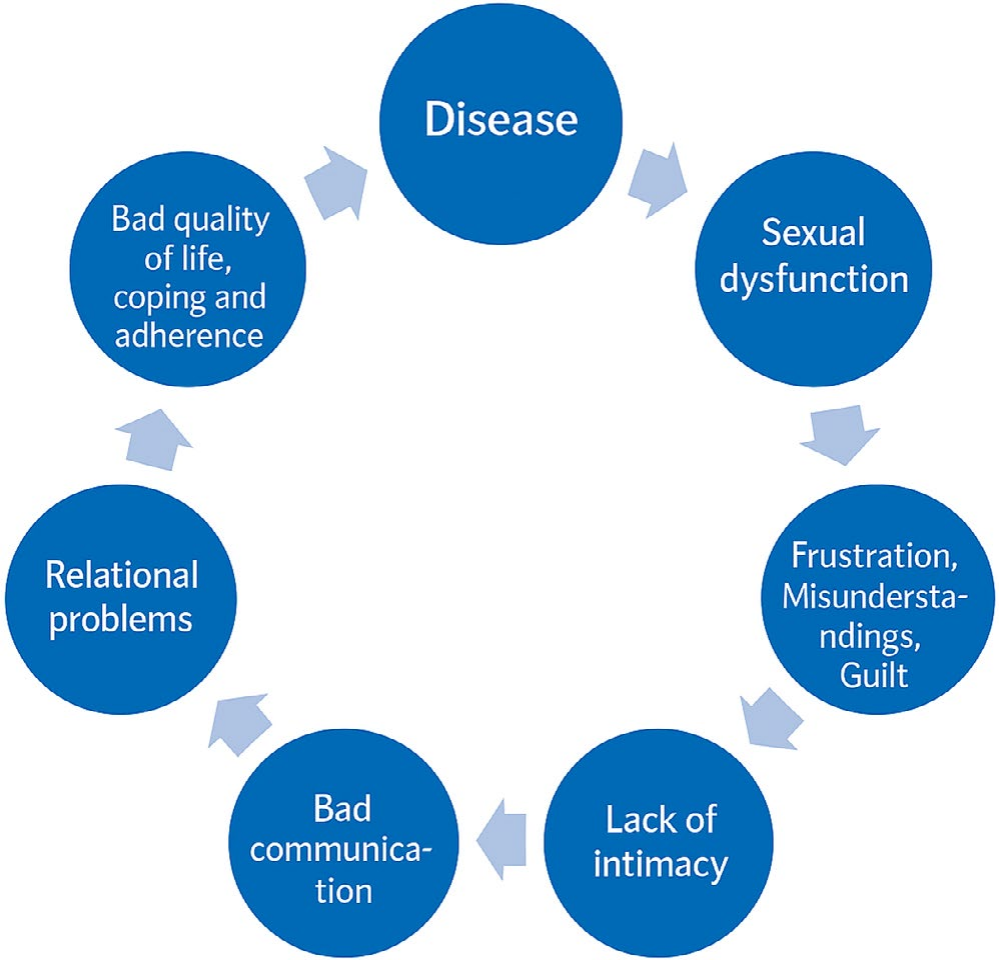
The vicious cycle of sexual dysfunction and health [19].

Sexuality is a crucial aspect of a person's identity, significantly affecting quality of life and overall well‐being [19, 20]. From birth to death, sexuality remains a vital component of human identity, playing a pivotal role in our lives. Sexual desires and behaviours shape our experiences and contribute to our self‐definition, impacting us in both positive and negative ways. As a fundamental aspect of being human, sexuality varies from person to person. It is a diverse and evolving element of our personal narratives. While sexuality can be shared with others, it ultimately remains a deeply personal experience [19, 20]. Given that sexuality is intertwined with individuals' identity, it cannot be separated from the person. This indicates that our sexuality always remains with us, even during treatment, hospitalisation or rehabilitation programmes. Illness and treatment can lead to sexual dysfunctions, but as described by the WHO, healthy sexuality is a dynamic equilibrium, where dysfunctions and inability to have penetrative sex are balanced by the patient's ability to cope with challenges, dysfunctions and crises. Sexual health—like health in general—is always contextual and multifactorial requiring holistic care that considers alle aspects of the patient's life [13, 20]. Sexual dysfunction resulting from illness can lead to a vicious circle (Figure 1), whereby untreated sexual complications gradually spiral into destructive feelings of shame, guilt, relational misunderstandings, dismissed self‐confidence, reduced quality of life and a loss of existential power [19, 21]. If nurses acknowledge that illness and treatment can cause sexual dysfunctions and support patients in managing these challenges, the vicious circle can be broken.

Additionally, sexual interactions can provide a sense of sanctuary during a tumultuous situation, and close relationships may foster purpose and unity amidst disorder and confusion that often characterise the daily lives of individuals living with cancer and chronic illnesses [19]. A systematic review confirmed associations between sexual health, health and well‐being [22]. Their findings highlight significant positive associations between sexual health, lower levels of depression and/or anxiety, and life satisfaction in diverse populations, including men and women, older adults, pregnant women, and individuals in same‐sex and mixed‐sex relationships. Studies on quality of life and health‐related quality of life consistently demonstrate significant positive associations with sexual health (Ibid). Research shows that sexuality is often not considered an essential aspect of fundamental care and is frequently overlooked in the healthcare system [9, 23]. Both nurses and patients rarely initiate conversations about sexuality due to numerous barriers, despite patients expressing the need to communicate with nurses about their sexual health and desiring nurses to initiate these conversations [5, 6, 9, 24, 25, 26]. Communication about sexuality is often perceived as difficult and tabooed [9, 27, 28, 29]. It can manifest as a ‘two‐way taboo’, where neither healthcare professionals nor patients initiate conversations about patient sexuality, resulting in deficient or non‐existent conversations [19].

From the patients' perspective, reasons for not discussing sexuality include a lack of trust or rapport with healthcare professionals and a belief that healthcare professionals do not have the time to discuss sensitive topics like sexuality. Patients also feel uncomfortable or embarrassed about discussing sexuality with healthcare professionals [9, 24, 30, 31]. Common barriers for nurses' initiating conversations about patients' sexual concerns include lack of education, knowledge, training and communication skills, as well as embarrassment, and the perception that sexual health is not part of their professional responsibility. Furthermore, sociocultural norms, beliefs, upbringing, lack of routine inquiry, insufficient priority, time constraints, absence of guidelines and organisational support are documented barriers [10, 16, 17, 18, 24, 32]. Fundamental nursing care related to sexuality extends beyond treating sexual dysfunction and providing information; it aims to initiate a constructive dialogue, whereby nurses catalyse changes through curiosity, empathy and respect. Ideally, regarding sexuality, it is about encouraging and empowering patients to reflect on their limitations and possibilities, thereby fostering relational communication and preventing misunderstandings, vicious circles, self‐censorship and diminished well‐being [19]. Despite an increase in research demonstrating patients' request for nurses to address sexual dysfunction from both a physical and psychosocial perspectives, only few models and frameworks within the nursing profession have incorporated this fundamental need as a core responsibility of the nurse. Therefore, continued focus on the importance of addressing sexuality in patient care is warranted. One of the more recently developed nursing frameworks is the Fundamentals of Care (FoC) framework [33]. The FoC framework provides a comprehensive lens through which the complexity of nursing care can be understood and addressed. By emphasising the integration of physical, psychosocial and relational dimensions of care, FoC highlights the multifaceted nature of nursing practice and supports a person‐centred approach, enabling nurses to address patients' sexuality alongside other fundamental care needs. Utilising the FoC framework may better prepare nurses to acknowledge and address sexuality as a fundamental aspect of patient care and support patients in articulating their sexual concerns.

## 3 Aim

The aim is to discuss how the FoC framework can support nurses in acknowledging and addressing sexuality as a fundamental aspect of patient care.

## 4 The Fundamentals of Care Framework

**FIGURE 2:**
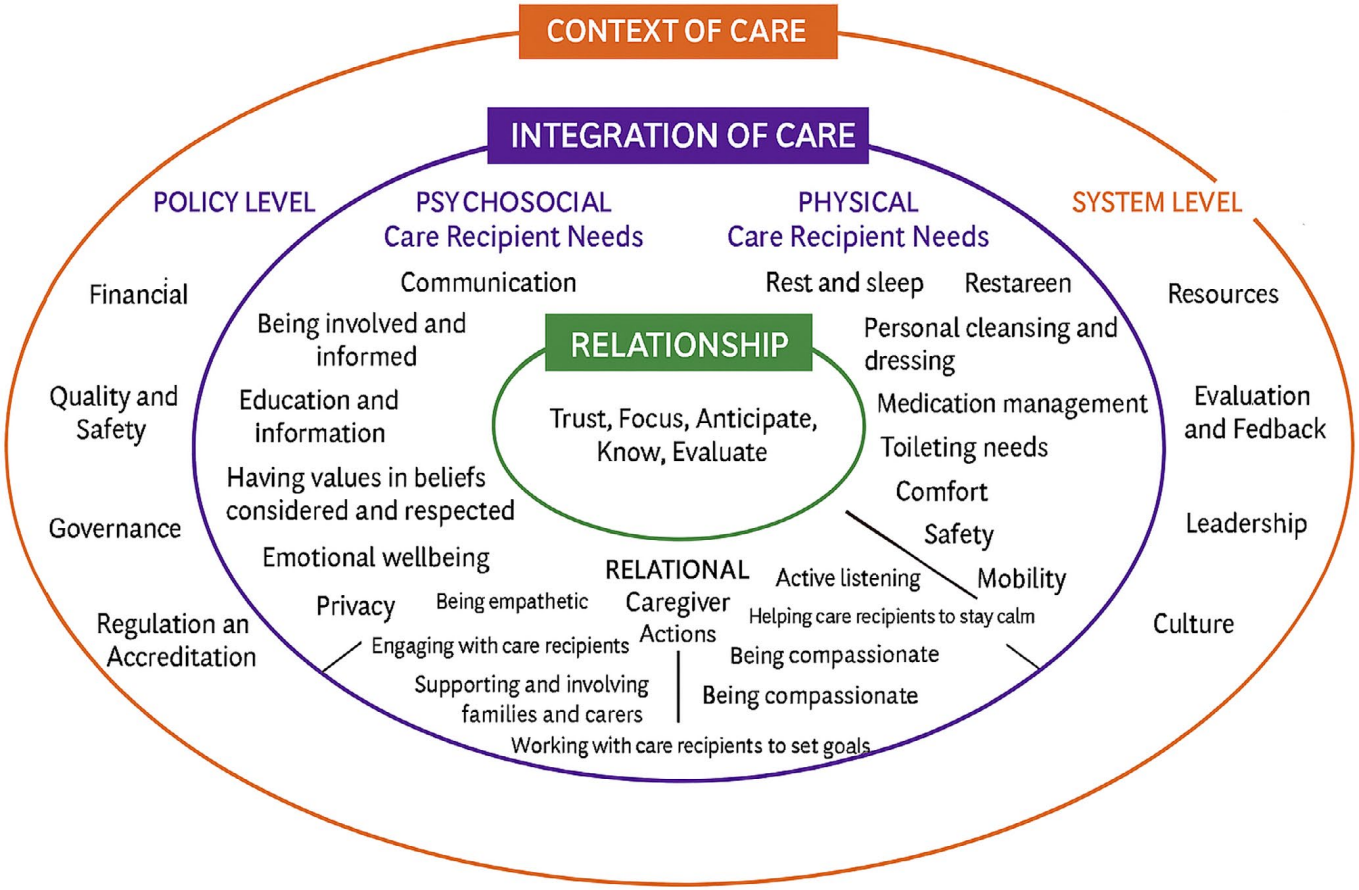
The Fundamentals of Care Framework [34, 35].

The FoC framework is a dynamic and internationally developed framework outlining what is involved in the delivery of safe, effective and high‐quality person‐centred care [33]. The FoC framework presents what concepts and elements are required for the delivery of high‐quality holistic care (Figure 2). Even though sexuality is a fundamental human need and an important aspect of existence, sexuality is not an explicitly included element in the current version of the FoC framework (Figure 2). The FoC framework has been developed based on a structured process in which elements evident in the framework have been generated from literature and research on fundamental nursing care. The first step in the structured process was to conduct a meta‐narrative review, where the purpose was to systematically uncover definitions of fundamental nursing and how aspects of nursing care were described in nursing literature [36]. The review resulted in a list that was subsequently categorised into 13 elements within nursing care. One of the 13 elements was ‘expressing sexuality’; however, this element was not included in the subsequent development of the framework in 2013 [34, 36]. Even though the framework has been refined three times since, sexuality remains absent in the FoC framework.

The FoC framework consists of three core dimensions: (1) relationship, (2) integration of care and (3) context of care (Figure 2).

The innermost dimension of the framework highlights elements central to the establishment of a relationship between the patient and the nurse, such as trust, focus, anticipation, knowledge and evaluation (Figure 2). The dimension underscores that establishing a relationship with the patient is a prerequisite for providing nursing care. The relationship is grounded in the nurse's commitment to care for the patient and significant others. This commitment also includes the responsibility to communicate relevant patient information to other healthcare professionals, caregivers and family members. Such commitment and communication ensure consistency and continuity of care as the patient moves from dependency to independence in self‐care activities or relies on others when independence is not achievable.

The second dimension focuses on integrating physical, psychosocial and relational elements in all care situations. The dimension builds on the nurse's initial assessment (see core dimension in Figure 2) and guides a series of practical actions addressing fundamental care needs. These include physical elements of care such as keeping the patient clean, warm, fed, hydrated, dressed, comfortable, mobile and safe, as well as psychosocial elements of care including keeping calm and coping, feeling respected, involved, informed and dignified. The physical and psychosocial elements are mediated through the relationship between the nurse and the patient. Key characteristics of these relationships are the ability of the nurse and patient to set mutual goals when addressing relevant fundamental care needs and for the nurse to demonstrate empathy, respect, compassion, consistency and continuity.

The third dimension (Figure 2) reflects the context of nursing care and presents the elements at a policy and system level that affect the delivery of high‐quality care. This includes factors such as resource availability, staffing levels, leadership and policy or regulatory frameworks—all of which can influence the quality of care and the strength of the nurse–patient relationship.

## 5 Methods

In this discursive paper, based on research and literature on sexuality as a fundamental need, we critically reflect on whether and how the Fundamentals of Care Framework may support nurses in acknowledging that sexuality is a fundamental care need and how it may support them in addressing the patient's needs regarding dysfunctional sexuality [37]. We conducted a broad, structured search in the following databases: PubMed, CINAHL and Embase, using the following keywords: sexuality, patients' sexuality, fundamental care need, holistic care, nursing theories, and selected state‐of‐the‐art literature on sexuality, sexual dysfunctions in patients and the nurse's role in addressing sexuality in patient care from the year 2010 to 2026. The literature search was restricted to studies published from 2010 onwards to ensure relevance to contemporary healthcare and nursing practice. Since 2010, a strengthened professional, educational and health policy discourse has emerged that explicitly positions sexuality as a legitimate and integral component of health and quality of life, rather than a private or peripheral concern. After 2010, sexuality became increasingly embedded within holistic health perspectives and professional ethical standards, rendering earlier literature less aligned with current conceptualisations of nursing care. We systematically reflected on the nurse's role in holistic care using the FoC framework as a tool to support nurses in addressing sexuality in their patient care.

## 6 Findings and Discussion

Building on the argument that sexuality is a fundamental need, this section discusses how the FoC framework can support nurses in acknowledging and addressing sexuality in patient care. The discussion emphasises key components of the three dimensions in the framework: relationship, integration of care and context of care, and draws on the identified literature on sexuality and sexual dysfunctions in patients and the nurse's role in acknowledging and addressing sexuality in patient care.

### 6.1 Relationship

**FIGURE 3:**
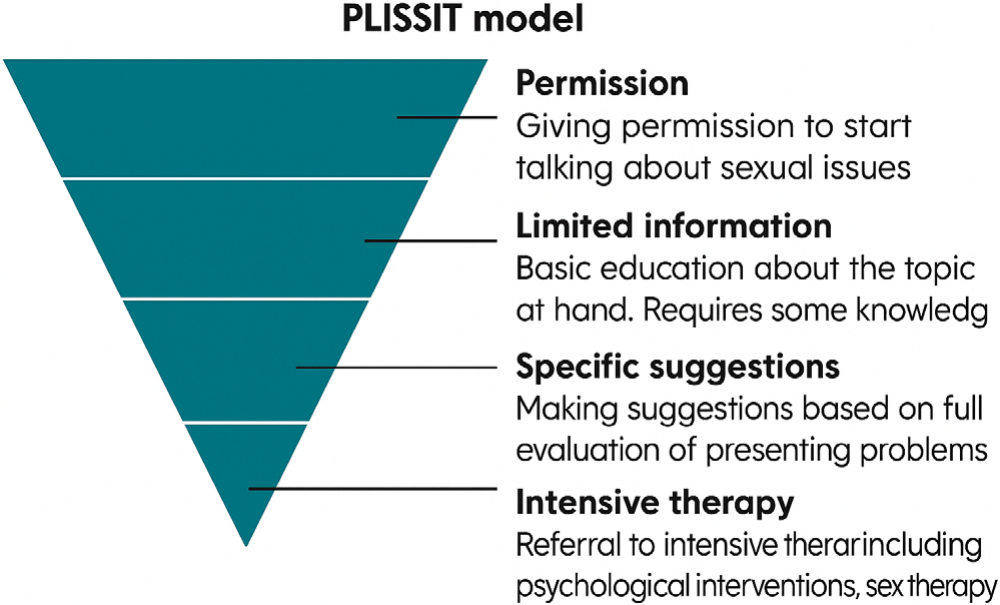
The PLISSIT model [20].

The nurse–patient relationship is central to the FoC framework and the foundation upon which high‐quality fundamental care is built. According to the FoC framework, establishing a positive relationship with a patient requires five elements: developing trust with the patient, focusing and giving the patient undivided attention, anticipating their needs, knowing enough about the patient to act appropriately, and evaluating the quality of the relationship [38]. As patients' sexuality is a taboo in nursing care and in society in general, many patients are embarrassed and uncomfortable initiating conversations about their sexuality with the nurse [9]. Thus, it is essential that the nurse takes on the responsibility for initiating this conversation. By initiating the conversation, the nurse helps break the two‐way taboo, which is important because studies show that patients fear being rejected or ignored if they initiate the conversation (ibid). According to the FoC framework, it is essential that nurses respect and focus on patients' essential needs. These needs are met by establishing a trusting nurse–patient relationship, thereby enabling patients to bring up sensitive topics such as sexuality. However, the FoC framework outlines what is involved in the delivery of high‐quality fundamental care; yet it does not provide concrete, evidence‐based guidance for practice in specific situations. Therefore, there is a need to draw on other theories, research and models to inform how to initiate conversations with patients about sexuality and signal that sexuality is a permissible topic. One well‐documented and widely recognised model in sexology that, through a structured approach, ensures that patients' sexuality is addressed is the PLISSIT Model (Figure 3) [20]. The PLISSIT Model is a framework used in the field of sexology to determine the different levels of intervention for individual patients. The letters of the name refer to the four different levels of intervention: Permission (P), Limited Information (LI), Specific Suggestions (SS) and Intensive Therapy (IT) (ibid). A meta‐analysis shows that sexual treatment based on the PLISSIT Model provides significant improvements in sexual function and ‘sexual and communication satisfaction’ [38]. The PLISSIT Model helps nurses initiate conversations about sexuality, identify sexual problems in collaboration with the patient and begin treatment through general information and/or specific suggestions. In some cases, more intensive therapy may be needed. The entire process is carried out in close collaboration with the patient, with ongoing reflection and evaluation to ensure that the focus remains on the patient's needs and that no boundaries are crossed. As mentioned, the PLISSIT Model helps nurses open the conversation by signalling to the patient that sexuality is a valid topic. Standard phrases can be helpful in inviting the patient to talk about any dysfunction they may be experiencing. For example: ‘We know from other patients with your condition that erectile dysfunction can occur—is this something you are experiencing?’ Such phrasing provides patients with an opening to share and helps nurses approach the topic in a concrete and supportive manner. The PLISSIT Model and the FoC framework can support nursing in ensuring that patients' fundamental needs regarding sexuality are not overlooked by opening the conversation and taking the patient's need for help with sexological issues seriously. If the nurse does not acknowledge the patient's needs or rejects the patient's desire for help, the patient may feel rejected. Being rejected or ignored can produce feelings of shame and embarrassment, which, according to social psychologist Thomas J. Scheff and sociologist Erving Goffman, signals a threat to the social bond and interaction [9]. Further, according to Kari Martinsen, Doctor of Philosophy, rejection can destroy or break trust, which is the essential part of relationships between the nurse and the patient [39]. When feeling ignored and not supported by the nurse, patients can become more vulnerable and embarrassed, inhibiting the establishment of a good relationship [9]. The FoC framework takes this into consideration by outlining that the relationship starts through establishing trust—the nurse needs to be trustworthy, and once trust is established [38], the nurse can focus on the patient to establish an understanding of their needs, including needs related to sexuality, and to assess the situation. Following this, the nurse must be able to anticipate patients' fundamental needs to ensure patients' well‐being and that they are and feel physically, psychosocially and emotionally safe [38]. This applies regardless of sexual orientation and gender identity. However, studies show that LGBT+ individuals experience uncertainty, lack of knowledge and insufficient education among healthcare professionals as barriers to receiving appropriate care. In addition to uncertainty and ignorance, informants in a Danish qualitative study reported the existence of cis‐ and heteronormative habitual thinking as a significant barrier to constructive dialogue [40]. A theoretical analysis conducted in the United States similarly indicates that employing Watson's Theory of Caring as a guiding framework for educational initiatives may strengthen attention to the sexual and reproductive health care needs of LGBT+ individuals, thereby supporting its integration into nursing education curricula [41].

### 6.2 Integration of Care

The second dimension of the FoC framework (Figure 2) focuses on how patients' individual fundamental care needs are addressed and the importance of the nurse–patient relationship in recognising and managing these complex needs [38]. What is important here is for the nurse to integrate the physical, psychosocial and relational needs of the patient in all situations of care to ensure person‐centred care [34]. As sexuality is a biopsychosocial phenomenon [12, 19], the nurse must integrate not only the physical elements of the patient's sexuality but also the psychological and relational elements. The PLISSIT Model's LI (Limited Information) and SS (Specific Suggestions) can help ensure a structured approach for the information and treatment to be implemented by informing and teaching at the LI level, based on the patient's problem statement, and further at the SS level, in collaboration with the patient, implementing specific treatment offers, while the FoC framework can support the nurse in performing person‐centred care by addressing and integrating the individual patient's physical, psychosocial and relational needs based on the latest evidence in the field. For example, when caring for a man treated for prostate cancer with erectile dysfunction due to the treatment, the nurse should not only focus on the erectile dysfunction and the treatment with medication but also assess the psychological impact of not feeling like ‘a real man’. Men with prostate cancer often feel inadequate and insufficient and have a low sense of manhood, which can affect the relationship with their partner and other people in their social circle [3]. Similarly, it is essential to integrate needs when caring for a woman who has undergone radiotherapy for uterine or cervical cancer and subsequently becomes sterile. Sterility is the purely biological consequence, but women often feel less valuable, which affects their femininity and potentially has consequences for their relationship with their partners. Providing person‐centred care in these situations requires that the nurse has biological, psychological and social knowledge about sexuality, competence in communication and confidence in addressing the patient's sexuality [24]. In practice, this means that when nurses undertake a fundamental care activity, e.g., related to sexuality, they need to be mindful of the underlying evidence informing the physical action; maintain focus on and knowledge of how to ensure dignity and respect for the patient; and interact with the patient in a way that reflects empathy, care and compassion.

### 6.3 Context of Care

The final and outer dimension of the FoC framework refers to the context of care. The context of care acknowledges that systems and organisations in which nurses work play a crucial role in how nursing care is addressed and delivered. The system level includes resources (e.g., physical resources such as equipment and human resources), culture (the values and norms of the system), leadership (leadership styles, how roles are defined and supported, and how staff are mentored), and evaluation and feedback (processes at individual, team and organisational levels that provide constructive feedback) [38].

Patients' sexual health is affected by determinants not only at the individual level but also at more proximal social levels, including relationships, the social environment of the community and the larger societal context [42]. Studies suggest that if the organisation has clear goals and guidelines regulating work with sexual health, nurses will acknowledge and address patients' sexual problems in their nursing care [43]. This means that if an organisation has values and objectives illustrated through a person‐centred nursing framework, such as FoC, the nurse will inevitably have to address the patients' problems related to sexuality and thereby help to break down the two‐way taboo in the healthcare system. When patients are met by nurses who initiate a conversation about sexuality and show that, on their ward, sexuality is a natural part of care and treatment, the patient will feel invited to open up about concerns and dysfunctions [18]. The opposite applies when patients experience that their attempts to get help for their sexual concerns and dysfunctions are neglected and rejected by the nurse or their sexual orientation or gender identity is overlooked or, in the worst case, belittled [9, 40].

## 7 Conclusion

This article investigates how the Fundamentals of Care (FoC) framework can support nurses in acknowledging and addressing patients' sexuality as a fundamental aspect of care. The article highlights that sexuality is an essential biopsychosocial dimension of human existence, often overlooked in clinical practice despite patients' needs to discuss the topic with healthcare professionals. The article argues that the FoC framework's focus on relationship, integration of care and context can contribute to breaking down taboos surrounding sexuality. In particular, the importance of establishing a trusting relationship with the patient, where the nurse actively initiates the conversation about sexuality, is emphasised. The PLISSIT model (Permission, Limited Information, Specific Suggestions, Intensive Therapy) is proposed as a supplement to FoC, to provide nurses with a structured approach to managing patients' sexual challenges. Furthermore, it is stressed that a holistic approach, integrating the physical, psychological and relational aspects of sexuality, is crucial. Finally, the significance of organisational frameworks that support and prioritise sexuality as an integral part of care is underlined, including clear guidelines and education. The article concludes that a combination of the FoC framework, the PLISSIT model and a supportive organisational culture can contribute to ensuring that patients' sexual needs are acknowledged and met, thereby improving their quality of life and well‐being.

## Author Contributions

Birgitte Schantz Laursen and Philippa Rasmussen conceived the idea for the article, with all authors collaborating on the design and methodology, as well as jointly analysing and interpreting the data. The initial draft of the article was composed by Birgitte Schantz Laursen and Philippa Rasmussen, while Britt Laugesen and Siri Lygum Voldbjerg provided critical feedback on the earlier version of the manuscript. All authors reviewed and approved the final version of the manuscript.

## Funding

The authors have nothing to report.

## Conflicts of Interest

The authors declare no conflicts of interest.

## Data Availability

Data sharing not applicable to this article as no datasets were generated or analysed during the current study.
